# Gene expression profiling via bioinformatics analysis reveals biomarkers in laryngeal squamous cell carcinoma

**DOI:** 10.3892/mmr.2015.3701

**Published:** 2015-04-29

**Authors:** GUO-FANG GUAN, YING ZHENG, LIAN-JI WEN, DE-JUN ZHANG, DUO-JIAO YU, YAN-QING LU, YAN ZHAO, HUI ZHANG

**Affiliations:** 1 Department of Otolaryngology, Head and Neck Surgery, The Second Hospital of Jilin University, Changchun, Jilin 130041, P.R. China; 2 Department of Otolaryngology, Head and Neck Surgery, Tumor Hospital of Jilin Province, Changchun, Jilin 130012, P.R. China

**Keywords:** laryngeal squamous cell carcinoma, differentially expressed genes, functional enrichment analysis, protein-protein interaction network, microRNA

## Abstract

The present study aimed to identify key genes and relevant microRNAs (miRNAs) involved in laryngeal squamous cell carcinoma (LSCC). The gene expression profiles of LSCC tissue samples were analyzed with various bioinformatics tools. A gene expression data set (GSE51985), including ten laryngeal squamous cell carcinoma (LSCC) tissue samples and ten adjacent non-neoplastic tissue samples, was downloaded from the Gene Expression Omnibus. Differential analysis was performed using software package limma of *R*. Functional enrichment analysis was applied to the differentially expressed genes (DEGs) using the Database for Annotation, Visualization and Integrated Discovery. Protein-protein interaction (PPI) networks were constructed for the protein products using information from the Search Tool for the Retrieval of Interacting Genes/Proteins. Module analysis was performed using ClusterONE (a software plugin from Cytoscape). MicroRNAs (miRNAs) regulating the DEGs were predicted using WebGestalt. A total of 461 DEGs were identified in LSCC, 297 of which were upregulated and 164 of which were downregulated. Cell cycle, proteasome and DNA replication were significantly over-represented in the upregulated genes, while the ribosome was significantly over-represented in the downregulated genes. Two PPI networks were constructed for the up- and downregulated genes. One module from the upregulated gene network was associated with protein kinase. Numerous miRNAs associated with LSCC were predicted, including miRNA (miR)-25, miR-32, miR-92 and miR-29. In conclusion, numerous key genes and pathways involved in LSCC were revealed, which may aid the advancement of current knowledge regarding the pathogenesis of LSCC. In addition, relevant miRNAs were also identified, which may represent potential biomarkers for use in the diagnosis or treatment of the disease.

## Introduction

Laryngeal squamous cell carcinoma (LSCC) is the most common type of laryngeal cancer. It is able to spread to regional cervical lymph nodes, or to more distant tissues, for example the lung. Improvements in current therapies have resulted in an improved quality of life for patients with LSCC (1). However, survival rates have not been significantly improved, which identifies the need for a change in clinical approach, requiring novel biomarkers for diagnosis, prognostic assessment and drug design (2).

Certain achievements have been made in the identification of biomarkers of LSCC. Järvinen *et al* (3) performed high-resolution copy number and gene expression microarray analyses to identify 739 genes overexpressed in LSCC. Gajecka *et al* (4) reported that polymorphisms of CYP1A1, CYP2D6, CYP2E1, NAT2, GSTM1 and GSTT1 were associated with an increased risk of LSCC. HLA class I antigen downregulation was identified as a poor prognostic marker for LSCC, which may reflect the reduction in the extent of CD8(+) T cell infiltration in LSCC lesions (5). Overexpression of osteopontin enhances the proliferation and invasiveness of LSCC (6), suggesting that it may represent a potential therapeutic target.

MicroRNAs (miRNAs) are short regulatory RNAs that modulate gene expression at the post-transcriptional level, and are involved in the pathogenesis of numerous types of cancer (7). Multiple miRNAs have been associated with LSCC. Overexpression of miR-21 contributes to the malignant phenotype of LSCC via inhibition of BTG family member 2 (8). miR-203 inhibits the proliferation of laryngeal carcinoma cells by modulating their survival (9). In addition, let-7a (10), miR-16 (11) and hsa-miR-34c (12) also have roles in laryngeal carcinoma.

Microarray technology provides global patterns of gene expression and therefore facilitates biomarker discovery. Lian *et al* (13) investigated tumorigenesis and regional lymph node metastasis in LSCC, whereas the present study focused specifically on the discovery of biomarkers associated with tumorigenesis. In the present study, gene expression profiles of LSCC were analyzed with a variety of bioinformatics tools, including functional enrichment and network analyses, in order to identify novel potential biomarkers. Additionally, miRNAs targeting these genes were also predicted. The identification of such biomarkers may be useful in the diagnosis and/or treatment of LSCC.

## Materials and methods

### Gene expression data

A gene expression data set (accession number GSE51985), which included ten LSCC tissue samples and ten adjacent non-neoplastic tissue samples, was downloaded from the Gene Expression Omnibus (http://www.ncbi.nlm.nih.gov/geo) (13). Gene expression levels were measured using the Illumina HumanHT-12 V4.0 expression beadchip platform (Illumina, San Diego, CA, USA; http://www.ncbi.nlm.nih.gov/geo/query/acc.cgi?acc=GPL10558). Platform annotation files were also acquired.

### Pretreatment and differential analysis

According to the annotation files, probes were initially mapped into the genes. If more than one probe was mapped into a single gene, levels of the probes were averaged as the final expression level for the specific gene. Following normalization, differential analysis was performed between the ten LSCC tissues and corresponding adjacent non-neoplastic tissues using the Linear Models for Microarray Analysis package (limma; http://www.bioconductor.org/packages/release/bioc/html/limma.html) (14) of *R*. P<0.05 and |log2 (fold change)|>1 were set as the threshold levels for the identification of differentially expressed genes (DEGs).

### Functional enrichment analysis

Functional enrichment analysis facilitated the identification of altered biological functions. In the present study, functional enrichment analysis was performed on the DEGs using the Database for Annotation, Visualization and Integration Discovery, version 6.7 (DAVID; http://david.abcc.ncifcrf.gov/) (15), which is able to reveal enriched Gene Ontology (GO) terms; the Kyoto Encyclopedia of Genes and Genomes (KEGG) for pathways (16) and InterPro, version 34.0 for protein domains (17) based on the hypergeometric distribution. P<0.05 was set as the threshold.

### Construction of protein-protein interaction (PPI) networks

Proteins ‘work together’ to exert certain biological functions, and the genome-wide identification of PPIs represents a significant step in the elucidation of the underlying molecular mechanisms. In present study, PPI networks were constructed for the protein products using information from the Search Tool for the Retrieval of Interacting Genes/Proteins (STRING, version 9.1; http://string-db.org/) (18). Interactions with a score (i.e. required confidence) >0.4 were retained in the network.

The proteins in the network serve as the ‘nodes’, and each pairwise protein interaction is represented by an undirected link. The ‘degree’ of a node corresponds to the number of interactions of that particular protein. The most highly connected nodes (those of a high degree) were considered to be the network ‘hubs’.

### Module analysis of the network

The PPI networks and regulatory associations between miRNAs and target genes were combined and subsequently visualized with Cytoscape, version 2.6.3 (http://cytoscape.org/) (19). Functional modules of the network were explored using ClusterONE, version 1.0 (http://www.paccanarolab.org/clusterone), a Cytoscape software plugin (20). P<0.01 was set as the cut-off value.

### Prediction of miRNAs

The miRNAs regulating the identified DEGs were predicted using WEB-based Gene Set Analysis Toolkit, version 2.0 (WebGestalt; http://bioinfo.vanderbilt.edu/webgestalt/) (21). Count ≥2 and false discovery rate (false positive rate, multiple testing corrected P-value) <0.1 were set as the threshold values.

## Results

### DEGs in LSCC

According to the criteria outlined (|log2(fold change)|>1; P<0.05), a total of 461 DEGs were identified in LSCC, of which 297 were upregulated and 164 were downregulated.

### Functional enrichment analysis

GO and KEGG pathway enrichment analyses were applied to the up- and downregulated genes using DAVID tools. The results are presented in Tables I and II.

The top five gene clusters with enrichment scores >2 are listed in Table I. The following molecular functional terms were significantly over-represented amongst the upregulated genes: ATP binding (GO: 0005524), adenyl ribonucleotide binding (GO: 0032559), adenyl nucleotide binding (GO: 0030554), purine nucleoside binding (GO: 0001883), nucleoside binding (GO: 0001882), purine nucleotide binding (GO: 0017076), ribonucleotide binding (GO: 0032553), purine ribonucleotide binding (GO: 0032555) and nucleotide binding (GO: 0000166). Amongst the downregulated genes, structural constituent of ribosome (GO: 0003735) and structural molecule activity (GO: 0005198) were significantly enriched.

In addition, the cell cycle (hsa04110), proteasome (hsa03050) and DNA replication (hsa03030) pathways were significantly enriched in the upregulated genes, while ribosome (hsa03010) was significantly enriched in the downregulated genes.

### PPI networks of the DEGs

A PPI network was constructed for the protein products of the DEGs using STRING. Interactions with a combined score of >0.4 were included in the network.

**Figure 1:**
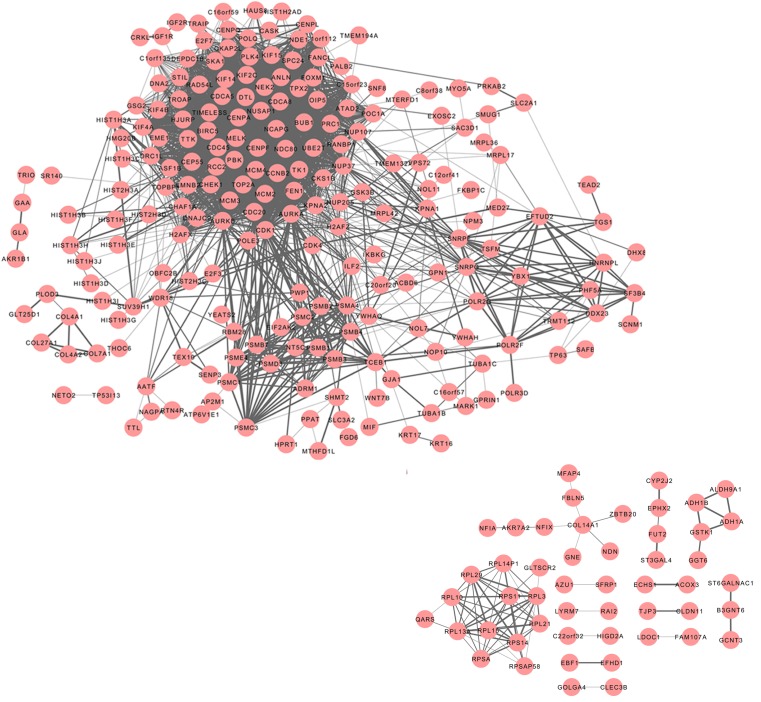
Protein-protein interaction networks for (A) upregulated genes and (B) downregulated genes in laryngeal squamous cell carcinoma. Red circles, protein products of differentially expressed genes; black lines, interactions between proteins. The thickness of the line is positively correlated with the degree of the interaction.

A network consisting of 213 proteins (nodes) and 2719 interactions (edges) was generated for the upregulated genes (Fig. 1). The top nodes, or hubs, characterized by a degree of >70, were aurora kinase B (AURKB), cyclin dependent kinase (CDK)1, cell division cycle (CDC) 20 homolog, AURKA, CDC45-like, budding uninhibited by benzimidazoles 1 homolog (BUB1), PDZ binding kinase (PBK), non-SMC condensin I complex subunit G (NCAPG), cell division cycle associated 8 (CDCA8), ubiquitin-conjugating enzyme E2T (UBE2T), minichromosome maintenance complex component 2 (MCM2), centromere protein F (CENPF), MCM4, NDC80 homolog kinetochore complex component (NDC80), CENPA, baculoviral IAP repeat-containing 5 (BIRC5), denticleless homolog (DTL), CHK1 checkpoint homolog (CHEK1), kinesin family member 2C (KIF2C), maternal embryonic leucine zipper kinase (MELK), topoisomerase (DNA) II α 170kDa (TOP2A) and cell division cycle associated 5 (CDCA5). Furthermore, a network comprised of 50 nodes and 80 edges was obtained for the downregulated genes. Few PPIs were observed amongst the downregulated genes, and therefore the subsequent analyses were focused on the upregulated genes.

### Module analysis

**Figure 2:**
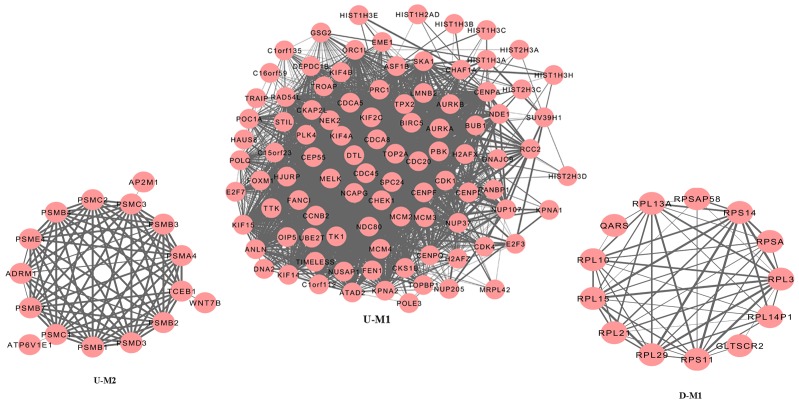
Modules identified from the protein-protein interaction networks. U-M1, Module 1 from the network of upregulated genes. U-M2, Module 2 from the network of upregulated genes. D-M1, Module 1 from the network of downregulated genes.

Module analysis was performed using ClusterONE to predict protein complexes, and the results are presented in Fig. 2. Two modules were identified amongst the upregulated genes: Module 1 (P<0.000) and Module 2 (P=5.559×10^−4^). Protein domain enrichment analysis was applied to the genes in the two modules using InterPro and the results are displayed in Table III. Serine/threonine protein kinase, active site (IPR008271); protein kinase, ATP binding site (IPR017441) and protein kinase, core (IPR000719) were significantly enriched amongst the genes from Module 1. No significantly enriched term was identified in the genes from Module 2. One module was generated by the downregulated genes (Fig. 2), but no significantly enriched protein domain was identified.

### miRNA-target interactions

**Figure 3:**
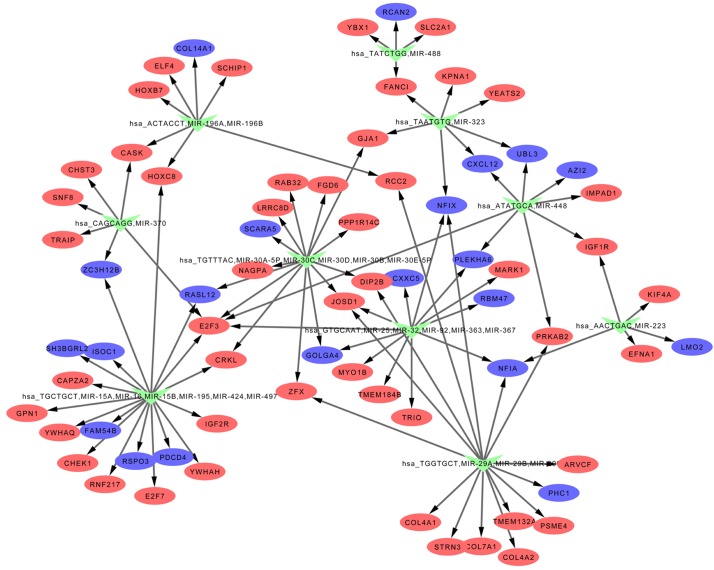
Gene regulatory network for the differentially expressed genes. Green triangles, micro RNAs; red ellipses, upregulated genes and blue ellipses, downregulated genes. The regulatory associations are represented by arrow lines.

miRNAs regulating the DEGs were predicted by WebGestalt, and the results are exhibited in Table IV and Fig. 3. hsa_GTGCAAT, miR-25, miR-32, miR-92, miR-363, miR-367, hsa_TGCTGCT, miR-15A, miR-16, miR-15B, miR-195, miR-424, miR-497, hsa_TGGTGCT, miR-29A, miR-29B and miR-29C were included in the list of identified miRNAs.

### Gene regulatory networks

The miRNA-target interactions were visualized by Cytoscape and thereby a gene regulatory network was established (Fig. 3).

## Discussion

Through the comparative analysis of gene expression data of LSCC and adjacent non-neoplastic control tissues, a total of 461 DEGs were identified in LSCC, of which 297 were upregulated and 164 were downregulated. Functional enrichment analysis indicated that the cell cycle, proteasome and relevant biological pathways were over-represented amongst the upregulated genes. Cell cycle progression is closely associated with multiple types of cancer (22–23), which indicated that the analysis results were of high confidence.

According to the results of previous studies, specific DEGs including CDK4, CDK1, MCM2, MCM3 and MCM4, have been implicated in LSCC (13,24). CDK4 is required for cell cycle G_1_ phase progression (25). Dong *et al* (26) reported that CDK4 was overexpressed in LSCC and suggested that it may have a critical role in cell proliferation together with cyclin D1. MCM2 has been implicated in the initiation of eukaryotic genome replication, which has been proposed as a marker of dysplasia and malignancy (27). Chatrath *et al* (28) reported aberrant expression of MCM2 in LSCC, while Torres-Rendon *et al* (29) indicated that MCM2 may be an indicator of growth and may provide a useful prognostic tool for oral epithelial dysplasia. MCM3 and MCM4 were also identified as DEGs in the present study. These findings were consistent with the results presented in a study by Lian *et al* (13).

Additional potential biomarkers were discovered in the present study. There is emerging evidence that glycogen synthase kinase (GSK)3β may be a tumor suppressor in oral cancer (30). It was therefore hypothesized that GSK3β may function in a similar way in LSCC. Several subunits of the proteasome were also identified in LSCC, including proteasome subunit β type 4 (PSMB4), PSMB7, PSMB1 and PSMC3. The ubiquitin-proteasome system is a critical regulator of cell growth and apoptosis (31), and proteasome inhibitors have been developed for use in cancer therapy (32–33). PSMB4 was identified as the first proteasomal subunit with oncogenic properties, promoting cancer cell survival and tumor growth *in vivo* (34). Elevated expression of PSMB4 is associated with poor prognosis in human cancer (34). PSMB7 was identified to be a prognostic biomarker in breast cancer (35). Therefore, further study of these subunits may reveal novel biomarkers for LSCC.

Network and module analyses were performed for the DEGs, and the identification of hub nodes and interactions may aid the elucidation of the underlying molecular mechanisms. AURKA and AURKB had a high degree in the PPI network for upregulated genes. Overexpression and hyperactivation of AURKA and AURKB have major roles in tumorigenesis, and therefore their inhibitors are already regarded as promising therapeutics for various types of cancer (36), including head and neck squamous-cell carcinoma (37). MCM2 and MCM4 also had a degree of >70, confirming their significant roles in the pathogenesis of LSCC.

Considering that miRNAs are closely involved in multiple types of cancer, a number of miRNAs targeting the DEGs were predicted by WebGestalt, including miR-15, miR-16, miR-25 and miR-195. Wu *et al* (11) reported that miR-16 was upregulated in LSCC and that it targets zyxin and promotes cell motility in human laryngeal carcinoma cell line HEp-2. Other miRNAs have been reported to have roles in various types of cancer. miR-15a forms a cluster with miR-16 at the chromosomal region 13q14, and functions as a putative tumor suppressor by targeting the oncogene BCL2 (38,39). miR-25 regulates apoptosis by targeting Bim in human ovarian cancer (40) and miR-195 is regarded as a predictor of poor prognosis in adrenocortical cancer (41). Future studies of these miRNAs may better describe the regulatory mechanisms underlying LSCC.

In conclusion, a number of key genes in LSCC were identified, which may represent novel biomarkers for diagnosis, prognosis and therapy. In addition, relevant miRNAs were also explored, and these may offer therapeutic targets for the modulation of abnormal gene expression in LSCC.

## Figures and Tables

**Table I:** GO enrichment analysis result for up- and downregulated genes in laryngeal squamous cell carcinoma.

Category	Term	Count	P-value
Upregulated genes			
Cluster 1	Enrichment score: 14.33726443940616		
BP	GO: 0007049~cell cycle	63	3.98×10^−27^
BP	GO: 0000278~mitotic cell cycle	46	4.67×10^−27^
BP	GO: 0022402~cell cycle process	50	8.03×10^−23^
BP	GO: 0000279~M phase	37	3.88×10^−20^
BP	GO: 0000280~nuclear division	30	1.39×10^−18^
BP	GO: 0007067~mitosis	30	1.39×10^−18^
BP	GO: 0000087~M phase of mitotic cell cycle	30	2.30×10^−18^
BP	GO: 0048285~organelle fission	30	4.23×10^−18^
BP	GO: 0022403~cell cycle phase	38	1.02×10^−17^
CC	GO: 0043232~intracellular non-membrane-bounded organelle	91	1.87×10^−15^
CC	GO: 0043228~non-membrane-bounded organelle	91	1.87×10^−15^
BP	GO: 0051301~cell division	29	3.35×10^−14^
CC	GO: 0005819~spindle	21	1.24×10^−13^
CC	GO: 0015630~microtubule cytoskeleton	35	4.61×10^−12^
CC	GO: 0044430~cytoskeletal part	41	6.61×10^−09^
BP	GO: 0007017~microtubule-based process	20	2.68×10^−08^
CC	GO: 0005856~cytoskeleton	49	6.72×10^−08^
CC	GO: 0005815~microtubule organizing center	17	2.28×10^−06^
CC	GO: 0005813~centrosome	16	2.37×10^−06^
CC	GO: 0005874~microtubule	14	3.79×10^−04^
Cluster 2	Enrichment score: 10.562682054877438		
CC	GO: 0031981~nuclear lumen	64	11×10^−14^
CC	GO: 0070013~intracellular organelle lumen	69	3.57×10^−13^
CC	GO: 0031974~membrane-enclosed lumen	70	8.50×10^−13^
CC	GO: 0043233~organelle lumen	69	1.07×10^−12^
CC	GO: 0005654~nucleoplasm	38	2.84×10^−08^
CC	GO: 0005730~nucleolus	29	4.12×10^−06^
Cluster 3	Enrichment score: 9.848444253761741		
CC	GO: 0005694~chromosome	34	1.72×10^−13^
CC	GO: 0000775~chromosome, centromeric region	19	7.04×10^−13^
CC	GO: 0044427~chromosomal part	30	1.90×10^−12^
CC	GO: 0000793~condensed chromosome	17	1.60×10^−10^
CC	GO: 0000779~condensed chromosome, centromeric region	12	5.30×10^−09^
CC	GO: 0000777~condensed chromosome kinetochore	11	1.91×10^−08^
CC	GO: 0000776~kinetochore	11	3.10×10^−07^
Cluster 4	Enrichment score: 7.286973695097799		
MF	GO: 0005524~adenosine triphosphate binding	52	9.82×10^−09^
MF	GO: 0032559~adenyl ribonucleotide binding	52	1.54×10^−08^
MF	GO: 0030554~adenyl nucleotide binding	53	3.18×10^−08^
MF	GO: 0001883~purine nucleoside binding	53	5.25×10^−08^
MF	GO: 0001882~nucleoside binding	53	6.58×10^−08^
MF	GO: 0017076~purine nucleotide binding	59	8.65×10^−08^
MF	GO: 0032553~ribonucleotide binding	57	1.19×10^−07^
MF	GO: 0032555~purine ribonucleotide binding	57	1.19×10^−07^
MF	GO: 0000166~nucleotide binding	65	1.29×10^−07^
Cluster 5	Enrichment score: 5.683294675111861		
BP	GO: 0007017~microtubule-based process	20	2.68×10^−08^
BP	GO: 0000226~microtubule cytoskeleton organization	15	1.06×10^−07^
BP	GO: 0007051~spindle organization	7	7.88×10^−05^
BP	GO: 0007010~cytoskeleton organization	20	8.29×10^−05^
Downregulated genes			
Cluster 1	Enrichment score: 2.991494823613603		
BP	GO: 0006414~translational elongation	9	3.72×10^−07^
MF	GO: 0003735~structural constituent of ribosome	10	2.59×10^−06^
CC	GO: 0033279~ribosomal subunit	9	4.39×10^−06^
CC	GO: 0022626~cytosolic ribosome	7	2.80×10^−05^
CC	GO: 0005840~ribosome	10	2.81×10^−05^
BP	GO: 0006412~translation	11	7.45×10^−05^
CC	GO: 0015934~large ribosomal subunit	6	1.30×10^−04^
CC	GO: 0044445~cytosolic part	7	8.82×10^−04^
CC	GO: 0022625~cytosolic large ribosomal subunit	4	2.70×10^−03^
CC	GO: 0030529~ribonucleoprotein complex	11	4.49×10^−03^
MF	GO: 0005198~structural molecule activity	12	4.68×10^−03^

Cluster, functional cluster; enrichment score, score for a functional cluster reflecting clustering effect; BP, biological process; CC, cellular components; MF, molecular function; count, number differentially expressed in a specific pathway; GO, gene ontology.

**Table II:** Kyoto Encyclopedia of Genes and Genomes pathway enrichment analysis result for up-regulated genes in laryngeal squamous cell carcinoma.

Term	Count	P-value
Upregulated genes		
Hsa04110: Cell cycle	16	4.12×10^−09^
Hsa03050: Proteasome	11	6.84×10^−09^
Hsa03030: DNA replication	6	4.21×10^−04^
Hsa04114: Oocyte meiosis	8	3.54×10^−03^
Hsa03040: Spliceosome	8	7.43×10^−03^
Downregulated genes		
Hsa03010: Ribosome	8	5.59×10^−06^
Hsa00071: Fatty acid metabolism	4	4.19×10^−03^

**Table III:** Significantly over-represented protein domains in the genes from the Module 1 network of upregulated genes.

Term	Count	P-value
IPR008271: Serine/threonine protein kinase, active site	11	1.61×10^−06^
IPR017441: Protein kinase, ATP binding site	12	2.11×10^−06^
IPR000719: Protein kinase, core	12	3.25×10^−06^
IPR002290: Serine/threonine protein kinase	9	1.01×10^−05^
IPR017442: Serine/threonine protein kinase-related	10	1.46×10^−05^
IPR018525: DNA-dependent ATPase MCM, conserved site	3	4.52×10^−04^
IPR001208: DNA-dependent ATPase MCM	3	5.80×10^−04^
IPR019821: Kinesin, motor region, conserved site	4	6.66×10^−04^
IPR001752: Kinesin, motor region	4	6.66×10^−04^
IPR007125: Histone core	4	8.16×10^−04^
IPR009072: Histone-fold	4	1.39×10^−03^
IPR002119: Histone H2A	3	2.68×10^ss03^

ATP, adenosine triphosphate; MCM, minichromosome maintenance complex component.

**Table IV:** Predicted miRNAs regulating the DEGs.

miRNA	Count	C	O	E	R	rawP	adjP
Hsa_GTGCAAT, miR-25, miR-32, miR-92, miR-363, miR-367	12	308	12	3.42	3.51	0.0002	0.0123
Hsa_TGCTGCT, miR-15A, miR-16, miR-15B, miR-195, miR-424, miR-497	18	593	18	6.59	2.73	0.0001	0.0123
Hsa_TGGTGCT, miR-29A, miR-29B, miR-29C	15	515	15	5.72	2.62	0.0007	0.0287
Hsa_ACTACCT, miR-196A, miR-196B	7	143	7	1.59	4.41	0.0011	0.0338
Hsa_ATATGCA, miR-448	8	208	8	2.31	3.46	0.0024	0.0492
Hsa_TAATGTG, miR-323	7	158	7	1.75	3.99	0.0020	0.0492
Hsa_AACTGAC, miR-223	5	94	5	1.04	4.79	0.0040	0.0703
Hsa_TATCTGG, miR-488	4	62	4	0.69	5.81	0.0050	0.0724
Hsa_TGTTTAC, miR-30A-5P, miR-30C, miR-30D, miR-30B, miR-30E-5P	14	572	14	6.35	2.20	0.0053	0.0724
Hsa_CAGCAGG, miR-370	6	153	6	1.70	3.53	0.0075	0.0922

miRNA, micro RNA; DEGs, differentially expressed genes; miR, micro RNA; Count, number of DEGS regulated by a specific miRNA; C, number of reference genes in the category; O, number of genes in the gene set and also in the category; E, expected number in the category; R, ratio of enrichment; rawP, raw P-value calculated by WebGastalt; adjP, adjusted P-value.
